# Supplementary material for: The Effects of Insulin-Like Growth Factor I and BTP-2 on Acute Lung Injury
Source: Int J Mol Sci. 2021 May 15;22(10):5244. doi: 10.3390/ijms22105244 (PMC8170877; doi:10.3390/ijms22105244)
Supplement: Supplementary file 1 [file ijms-22-05244-s001.zip › ijms-22-05244-s001.pdf]

International Journal of *Molecular Sciences*

MDPI

Table S1. Primer list..

Gene	Forward	Reverse
TLR-4	GCCTTTCAGGGAATTAAGCTCC	GATCAACCGATGGACGTGTAAA
NFATC1	GGAGAGTCCGAGAATCGAGAT	TTGCAGCTAGGAAGTACGTCT
TRPC6	GCTTCCGGGGTAATGAAAACA	GTATGCTGGTCCTCGATTAGC
TRPC3	GCCTTCATGTTCGGTGCTC	GGTCACCTCCAGATGCTCATT
ORAI 1	CTCAACTCGGTCAAAGAGTCAC	CACGACCTCTGCTAGGAAAAG
CALCINEURIN	GTGAAAGCCGTTCCATTTCCA	GAATCGAAGCACCCTCTGTTATT
IL-1B	GAAATGCCACCTTTTGACAGTG	TGGATGCTCTCATCAGGACAG
IL-17	GGCCCTCAGACTACCTCAAC	TCTCGACCCTGAAAGTGAAGG
IL-6	CTGCAAGAGACTTCCATCCAG	AGTGGTATAGACAGGTCTGTTGG
TNF-A	CCTGTAGCCCACGTCGTAG	GGGAGTAGACAAGGTACAACCC
IFN-g	ATGAACGCTACACACTGCATC	CCATCCTTTTGCCAGTTCCTC
CD-31	CTGCCAGTCCGAAAATGGAAC	CTTCATCCACCGGGGCTATC
VEGF	GCACATAGAGAGAATGAGCTTCC	CTCCGCTCTGAACAAGGCT
A-SMA	GTCCCAGACATCAGGGAGTAA	TCGGATACTTCAGCGTCAGGA
SP-D	CTCCCACTATCAGAAAGCTGC	CCCACATCTGTCATACTCAGGAA
CASPASE 3	ATGGAGAACAACAAAACCTCAGT	TTGCTCCCATGTATGGTCTTTAC
NGAL	GGGAAATATGCACAGGTATCCTC	CATGGCGAACTGGTTGTAGTC
GAPDH	TGGCCTTCCGTGTTCCTAC	GAGTTGCTGTTGAAGTCGCA

Notes: TLR-4: Toll-like receptor 4, NFATC1: Nuclear factor of activated T cells 1; TRPC3: Transient receptor potential cation channel subfamily C member 3, TRPC6: Transient receptor potential cation channel subfamily C member 6, ORAI-1: ORAI calcium release-activated calcium modulator 1, IL-1B: Interleukin 1 beta, IL-17:Interleukin 17; IL-6: Interleukin-6; TNF-α:Tumor Necrosis Factor -α, IFN-g: Interferon Gamma, CD-31: Platelet/endothelial cell adhesion molecule 1, VEGF: Vascular endothelial growth factor, a-SMA: Alpha Smooth Muscle actin, NGAL: Lipocalin 2; GAPDH: Glyceraldehyde-3-Phosphate Dehydrogenase.
